# Pathophysiological background and prognostic implication of systolic aortic root motion in non-ischemic dilated cardiomyopathy

**DOI:** 10.1038/s41598-019-40386-z

**Published:** 2019-03-07

**Authors:** Matthias Aurich, Matthias Niemers, Patrick Fuchs, Sebastian Greiner, Matthias Müller-Hennessen, Lorenz Uhlmann, Evangelos Giannitsis, Philipp Ehlermann, Benjamin Meder, Hugo A. Katus, Derliz Mereles

**Affiliations:** 1 Department of Internal Medicine III, Cardiology, Angiology and Pneumology, University of Heidelberg, Heidelberg, Germany; 2 Institute of Medical Biometry and Informatics, University of Heidelberg, Heidelberg, Germany

## Abstract

Recordings of aortic root movement represent one of the first accomplishments of ultrasound in medicine and mark the beginning of functional cardiac imaging. However, the underlying mechanism is not completely understood. Since the aortic root is directly connected to the cardiac skeleton we hypothesize, that the amplitude of systolic aortic root motion (SARM) may be mainly caused by displacement of the cardiac base towards the apex and might therefore be used as measure of left ventricular longitudinal function (LV-LF). One hundred and eighty patients with dilated cardiomyopathy and 180 healthy controls were prospectively included into this study. SARM was lower in patients compared to controls (9 ± 3 mm vs. 12 ± 2 mm, p < 0.001) and lowest in patients with cardiovascular events (9 ± 3 mm vs. 7 ± 3 mm, p < 0.001). During a median follow-up time of 38 months, the combined end-point of cardiovascular death or hospitalization for heart failure was reached by 25 patients (13.9%). Reduced SARM had significant prognostic impact on outcome (hazard ratio 0.74, 95% confidence interval 0.63–0.88, p < 0.001) and remained an independent predictor in the multivariate analysis. Compared to parameters with potential influence on its mechanism, SARM correlated best (r = 0.75, p < 0.001) with global longitudinal strain (GLS). SARM may therefore represent an alternative echocardiographic parameter for the assessment of LV-LF, particularly when GLS is not feasible or apical views are not available.

## Introduction

Left ventricular (LV) contraction is determined by a complex arrangement of muscle fiber layers and comprises longitudinal shortening and axial twist. Impairment of the longitudinal component is often the first sign of LV dysfunction even when ejection fraction (EF) is still normal^1,2^. Beyond that diagnostic significance, LV longitudinal function (LF) has additive prognostic value when EF is already reduced^3^. Therefore, techniques that enable assessment of LV-LF are highly relevant and should nowadays complement every cardiac imaging report^4,5^.

Echocardiography is by far the most widely used imaging modality in cardiology. 2- and 3-dimensional systems have improved its diagnostic potential continuously^6,7^ but they still face limitations especially when dealing with poor acoustic windows. In such cases M-mode echocardiography is a helpful alternative. Due to its high temporal resolution movement of echogenic structures can easily be visualized even when image quality is reduced^8^.

The first description of moving ultrasound signals using M-mode echocardiography dates back to the early fifties when Edler assumed these patterns to originate from the anterior left atrial wall^9^. By contrast enhanced echocardiography using saline injection in the supravalvular position Gramiak *et al*. confirmed that undulating parallel signals medial to the mitral valve actually arise distal from the aortic valve and thus represent a portion of the aorta. Furthermore, they could demonstrate, that the pattern of motion obtained from the aortic root equals that of earlier M-mode recordings from the mitral ring^10,11^. Aortic root motion has subsequently been investigated as a surrogate parameter of left ventricular systolic and diastolic function.

**Figure 1:**
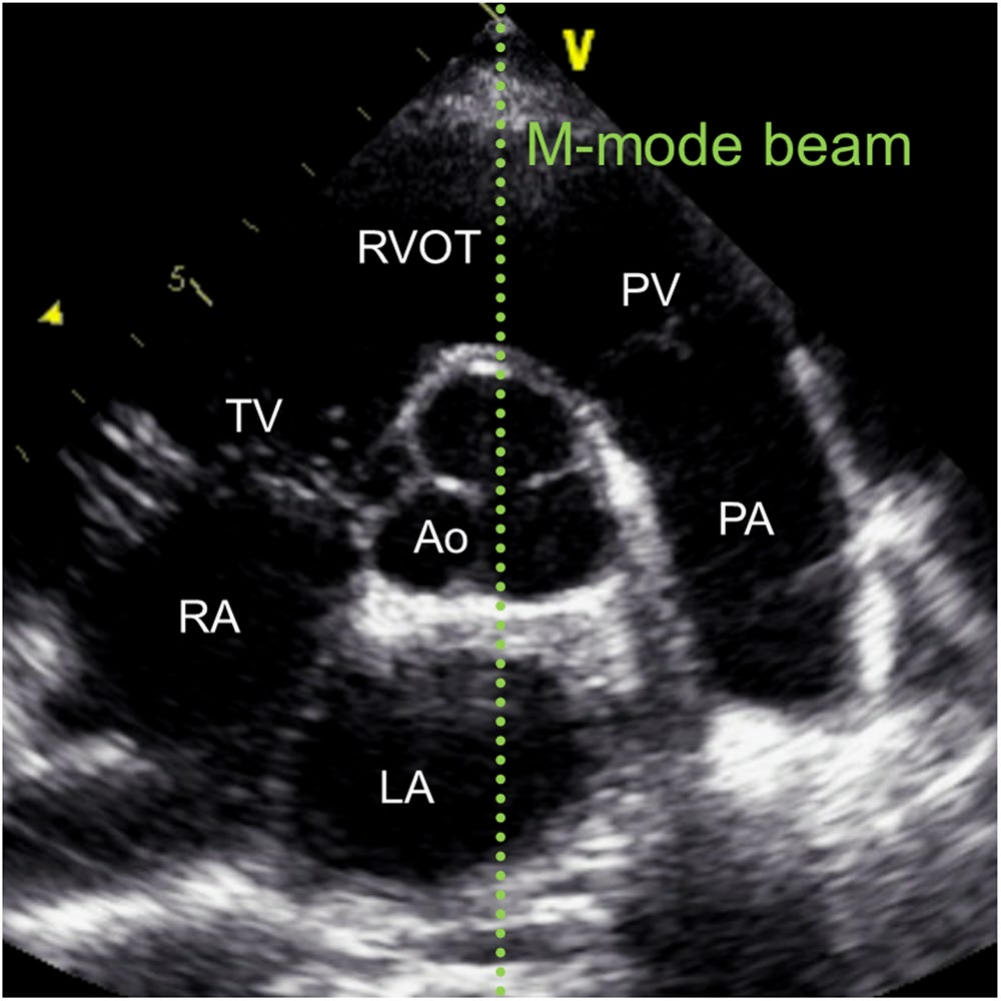
Parasternal echocardiographic B-mode image at the level of the valvular plane. The M-mode beam (light green) is directed through the center of the aortic root (Ao). LA, left atrium; PA, pulmonary artery; PV, pulmonary valve; RA, right atrium; RVOT, right ventricular outflow tract; TV, tricuspid valve

**Figure 2:**
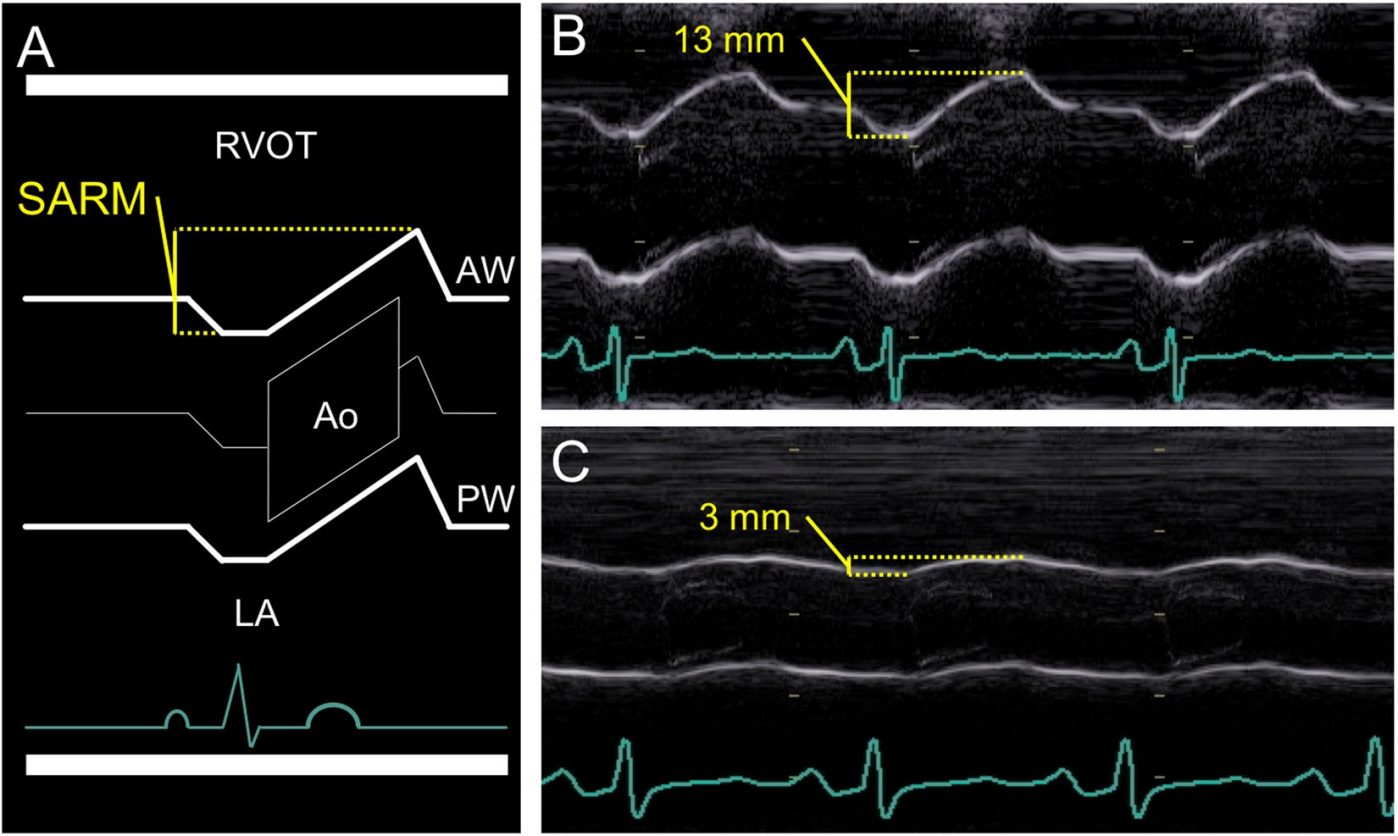
Left: Schematic representation of one cardiac cycle recorded by M-mode echocardiography at the level of the aortic root (Ao). Right: Two examples of SARM measurement in a healthy individual (**B**) and a patient with markedly depressed left ventricular longitudinal function (**C**). AW, anterior wall; LA, left atrium; PW, posterior wall; RVOT, right ventricular outflow tract

As part of the cardiac skeleton the aortic annulus and the attached aortic root follow the valvular plane displacement during the heart cycle. Therefore, we hypothesize, that the amplitude of systolic aortic root motion (SARM) obtained by M-Mode echocardiography may be used as a measure of global LV-LF (Figs 1 and 2).

## Results

### Characteristics of the study population

One hundred and eighty patients with dilated cardiomyopathy were matched by age and gender with 180 healthy control subjects. Median follow-up time was 1,150 days (38 months), 4 patients were lost to follow-up. Clinical, laboratory, and echocardiographic parameters are summarized in Table 1 and Table 2. Male subjects predominated in this study (n = 278 [77%]).

**Table 1:** Characteristics of patients and healthy controls.

Parameter	Patients (n = 180)	Controls (n = 180)	p-value
**Baseline**			
Male gender, n (%)	139 (77)	139 (77)	1
Age, years	56 (48;65)	58 (50;67)	0.191
BSA, m^2^	2.0 ± 0.2	1.9 ± 0.2	<0.001
BMI, kg/m^2^	27 (24;30)	25 (23;27)	<0.001
Heart rate, min^−1^	73 ± 18	62 ± 9	<0.001
BP systolic, mmHg	121 ± 18	136 ± 15	<0.001
BP diastolic, mmHg	75 ± 11	86 ± 9	<0.001
MAP, mmHg	91 ± 13	102 ± 10	<0.001
**Clinical chemistry**			
NT-proBNP, ng/L	489 (108;1,339)	55(30;97)	<0.001
hs-TNT, pg/mL	11 (6;24)	5 (4;7)	<0.001
**Echocardiography**			
IVS, mm	9 (8;10)	10 (9;11)	0.011
PW, mm	8 (7;9)	8 (7;9)	0.656
EDD, mm	57 ± 9	48 ± 4	<0.001
ESD, mm	45 (38;54)	34 (31;37)	<0.001
LV mass/BSA, g/m^2^	187 ± 64	140 ± 30	<0.001
EDV, mL	152 (117;209)	116 (93;137)	<0.001
ESV, mL	93 (67;149)	48 (38;57)	<0.001
EF, %	38 (26;44)	58 (56;61)	<0.001
MAPSE, mm	12 (8;15)	16 (14;17)	<0.001
MASV, cm/s	6 (5;9)	10 (8;11)	<0.001
GLS, %	−12.7 ± 4.8	−19.5 ± 1.7	<0.001
SARM, mm	9 ± 3	12 ± 2	<0.001
LA-Volume/BSA, mL/m^2^	37 (28;48)	26 (22;31)	<0.001
LA-VC, %	46 (29;54)	58 (52;64)	<0.001
E/A	1.0 (0.8;1.3)	1.1 (0.8;1.3)	0.577
E/e’	7 (5;9)	6 (5;7)	<0.001
E-DT, ms	197 (157;253)	209 (183;244)	0.239
SPVF/DPVF	1.1 ± 0.5	1.4 ± 0.4	0.005

BMI, body mass index; BP, blood pressure; BSA, body surface area; DPVF, diastolic pulmonary venous flow; E/A, ratio of mitral inflow velocity (E) to atrial contraction velocity (A); E/e’, ratio of mitral inflow velocity (E) to tissue Doppler mitral annular velocity (e’); E-DT, E-wave deceleration time; EDD, end-diastolic diameter; EDV, end-diastolic volume; EF, ejection fraction; ESD, end-systolic diameter; ESV, end-systolic volume; GLS, global longitudinal strain; IVS, interventricular septum; hs-TNT, high sensitive Troponin T; LA-VC, left atrial volume change; MAP, mean arterial pressure; MAPSE, mitral annular plane systolic excursion; MASV, mitral annular systolic velocity; NT-proBNP, N-terminal pro Brain natriuretic peptide; PW, posterior wall; SARM, systolic aortic root motion; SPVF, systolic pulmonary venous flow.

**Table 2:** Characteristics of patients stratified according to an event or no event.

Parameter	No event (n = 155)	Event (n = 25)	p-value
**Baseline**			
Male gender, n (%)	121 (78)	18 (72)	0.502
Age, years	55 ± 14	56 ± 15	0.940
BSA, m^2^	2.0 ± 0.2	1.9 ± 0.3	0.077
BMI, kg/m^2^	27 (24;30)	25 (27;29)	0.095
Heart rate, min^−1^	71 ± 19	79 ± 16	0.023
BP systolic, mmHg	122 ± 19	120 ± 15	0.748
BP diastolic, mmHg	75 ± 11	76 ± 10	0.758
MAP, mmHg	91 ± 13	91 ± 10	0.975
NYHA > II, n	19 (13)	7 (29)	0.040
**Clinical chemistry**			
NT-proBNP, ng/L	432 (89;1,164)	1,293 (662;3,934)	<0.001
hs-TNT, pg/mL	10 (6;22)	14 (8;48)	0.020
**Comorbidities**			
Hypertension, n (%)	80 (52)	15 (63)	0.351
Dyslipidemia, n (%)	41 (27)	8 (33)	0.506
Diabetes, n (%)	27 (18)	8 (33)	0.072
Renal dysfunction, n (%)	83 (58)	12 (52)	0.623
**Heart catheterization**			
LVEDP, mmHg	16 (12;24)	23 (13;31)	0.020
**Echocardiography**			
IVS, mm	9 ± 2	9 ± 2	0.971
PW, mm	8 (7;9)	7 (6;9)	0.365
EDD, mm	56 ± 8	60 ± 9	0.035
ESD, mm	45 ± 11	53 ± 9	0.004
LV mass/BSA, g/m^2^	88 (73;105)	101 (90;113)	0.012
EDV, mL	145 (116;203)	199 (153;267)	0.007
ESV, mL	88 (63;135)	147 (91;204)	<0.001
EF, %	39 (27;45)	24 (16;36)	<0.001
MAPSE, mm	12 (9;15)	8 (7;11)	<0.001
MASV, cm/s	7 (5;10)	5 (4;7)	<0.001
GLS, %	−13.2 ± 4.7	−9.4 ± 3.8	<0.001
SARM, mm	9 ± 3	7 ± 3	<0.001
LA-Volume/BSA, mL/m^2^	35 (27;48)	42 (35;48)	0.014
LA-VC, %	48 (32;55)	32 (21;45)	0.014
E/A	1.0 (0.8;1.2)	1.1 (0.8;2.4)	0.421
E/e’	7 (5;9)	10 (8;11)	<0.001
E-DT, ms	217 ± 71	169 ± 53	0.002
SPVF/DPVF	1.2 ± 0.5	1.0 ± 0.5	0.060

BMI, body mass index; BP, blood pressure; BSA, body surface area; DPVF, diastolic pulmonary venous flow; E/A, ratio of mitral inflow velocity (E) to atrial contraction velocity (A); E/e’, ratio of mitral inflow velocity (E) to tissue Doppler mitral annular velocity (e’); E-DT, E-wave deceleration time; EDD, end-diastolic diameter; EDV, end-diastolic volume; EF, ejection fraction; ESD, end-systolic diameter; ESV, end-systolic volume; GLS, global longitudinal strain; IVS, interventricular septum; hs-TNT, high sensitive Troponin T; LA-VC, left atrial volume change; LVEDP, left ventricular end-diastolic pressure; MAP, mean atrial pressure; MAPSE, mitral annular plane systolic excursion; MASV, mitral annular systolic velocity; NT-proBNP, N-terminal pro Brain natriuretic peptide; PW, posterior wall; SPVF, systolic pulmonary venous flow.

### Determining factors of SARM

**Figure 3:**
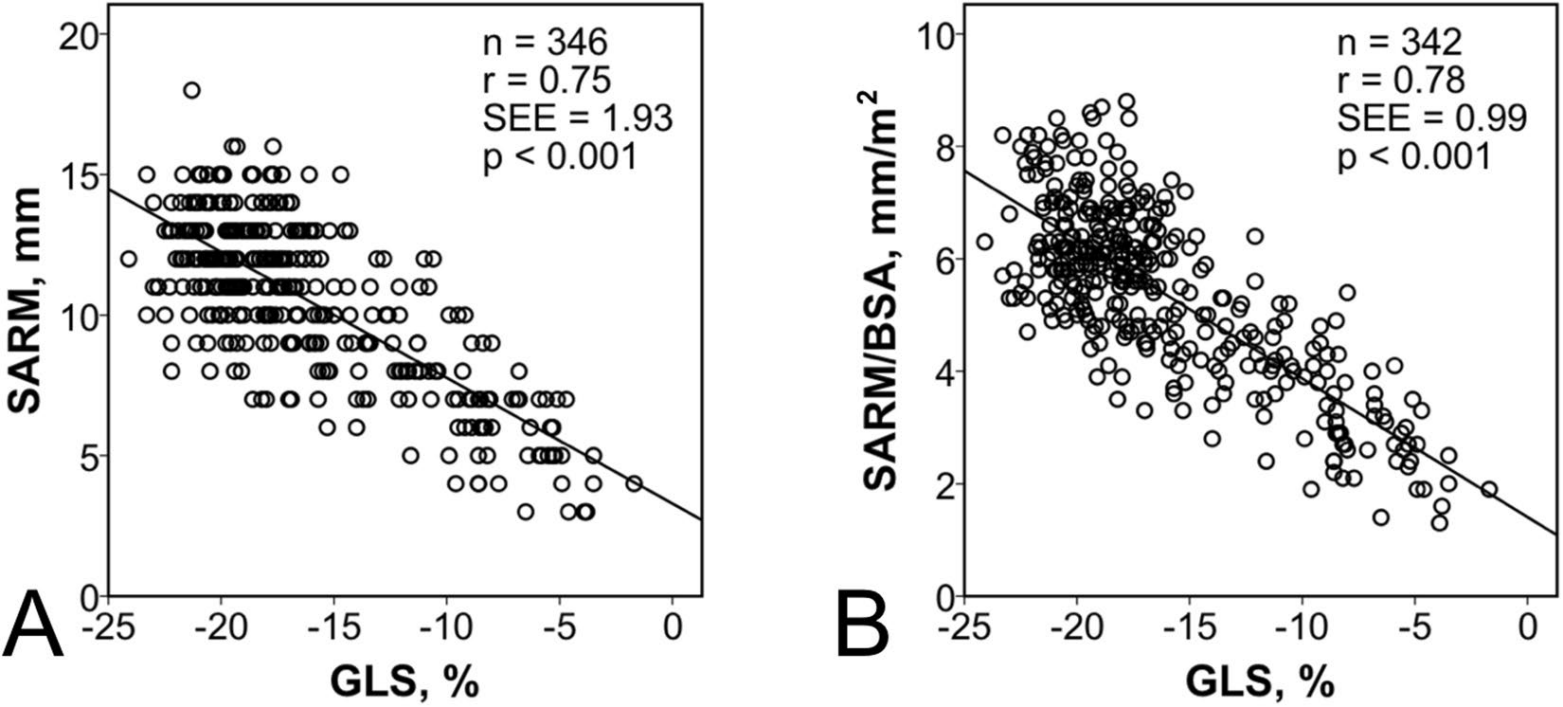
Correlations between systolic aortic root motion (SARM) and global longitudinal Strain (**A**) and SARM adjusted for body surface area (BAS) and GLS (**B**). SEE, standard error of estimate.

A potential association between different hemodynamic as well as functional cardiac parameters and SARM was tested by linear regression analysis. Results of correlation between SARM and global longitudinal strain (GLS), EF, LV stroke volume (SV), left atrial volume change (LA-VC) and mean arterial pressure (MAP) are presented in Table 3, Fig. 3 and Supplementary Fig. S1. Best correlations were found for SARM and GLS (r = 0.75 and 0.78, respectively, Fig. 3A,B) as well as SARM and EF (r = 0.74, Supplementary Fig. S1A). Weaker associations were found to SV and LA-VC (r = 0.57 and 0.61, respectively, Supplementary Fig. S1B,C) and no correlation to MAP (r = 0.21, Supplementary Fig. S1D).

**Table 3:** Linear regression analysis.

Parameter	Equation	r	SEE	p-value
SARM - GLS	f(x) = −0.5x + 3.3	0.75	1.93	<0.001
SARM/BSA - GLS	f(x) = −0.3x + 1.4	0.78	0.99	<0.001
SARM - EF	f(x) = 0.2x + 3.5	0.74	2.01	<0.001
SARM - SV	f(x) = 0.1x + 5.0	0.57	2.44	<0.001
SARM - LA-VC	f(x) = 0.1x + 4.3	0.61	2.38	<0.001
SARM - MAP	f(x) = 0.1x + 6.0	0.21	2.81	<0.001

BSA, body surface area; EF, ejection fraction; GLS, global longitudinal strain; LA-VC, left atrial volume change; MAP, mean arterial pressure; SARM, systolic aortic root motion; SEE, standard error of estimate; SV, stroke volume.

### SARM in patients and healthy controls

Total excursion of SARM was lower in the patient cohort compared with healthy individuals (9 ± 3 mm vs. 12 ± 2 mm, p < 0.001) and was even stronger diminished in patients with compared to patients without an event (7 ± 3 mm vs. 9 ± 3 mm, p < 0.001). The ability of SARM to differentiate patients with DCM from healthy subjects was tested by Receiver operating characteristic (ROC) analysis and Youden’s index and yielded a cutoff value of 11 mm (area under the curve [AUC] = 0.85, 95% confidence interval [CI] 0.82–0.89).

**Figure 4:**
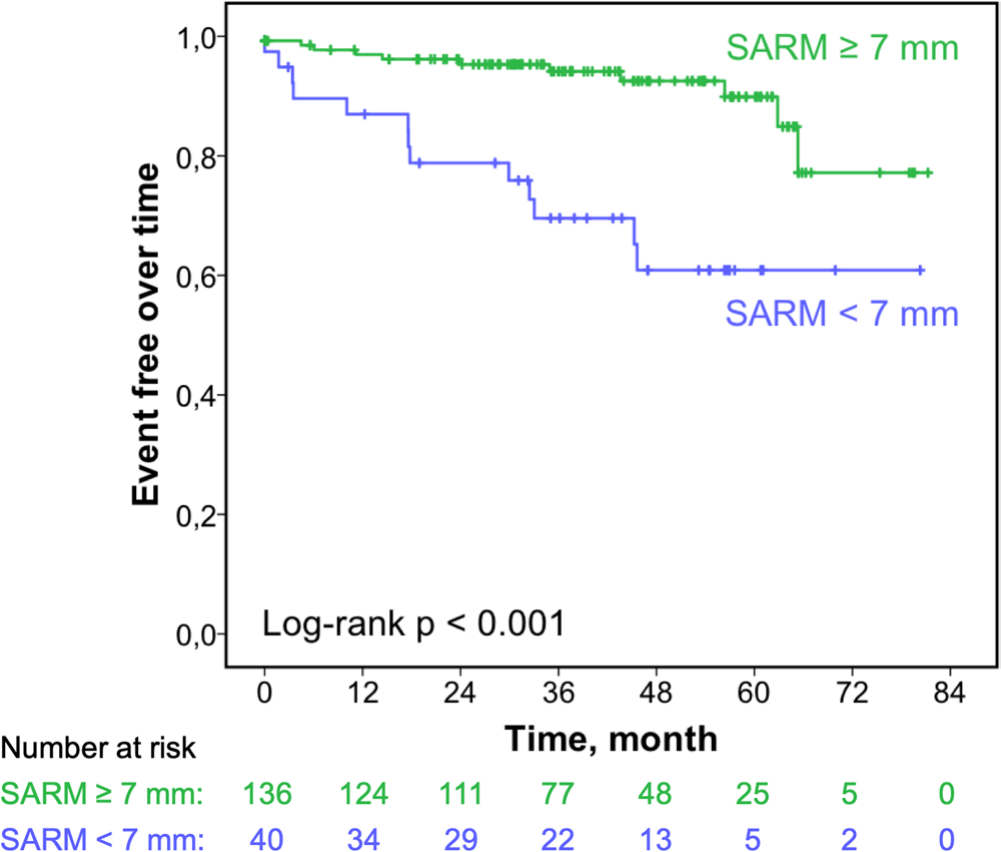
Kaplan-Meier curve displaying the frequency of cardiac events over time for a given cutoff value of systolic aortic root motion (SARM).

The combined end point of cardiovascular death or hospitalization for heart failure was observed in 25 patients, including 22 patients with acute heart failure with need for hospital admission and 3 cases of cardiovascular death. An optimal cutoff value for SARM to discriminate patients at risk for a cardiac event was found to be <7 mm calculated by ROC analysis and Youden’s index (AUC = 0.72, 95% CI 0.61–0.83). The frequency of cardiac events over time is displayed by Kaplan-Meier curves (Fig. 4), which were compared using the Log-rank test. Results of univariate Cox regression analysis are shown in Table 4. N-terminal pro–brain natriuretic peptide (NT-proBNP), New York Heart Association (NYHA) functional classes III and IV and Diabetes as clinical as well as LV end-diastolic pressure assessed by left heart catheterization as invasively determined parameter were associated with the occurrence of adverse events (p < 0.05 each). Among echocardiographic parameters EF and SARM had the highest impact on patient outcomes (p < 0.001 each). Based on the univariate Cox regression SARM was entered into a clinical Model including NT-proBNP, NYHA functional class III and IV and Diabetes (model 1, Table 5) and 2 echocardiographic models, the first consisting of LV longitudinal function parameters GLS, MAPSE and MASV (model 2, Table 5) and the second including EF and GLS as parameters of systolic LV function (model 3, Table 5). In the clinical model SARM remained independently predictive regarding cardiac death and hospitalization, whereas none of the longitudinal or systolic function parameters in both echocardiographic models was independently predictive.

**Table 4:** Univariate Cox regression analysis.

Parameter	HR	CI	p-value
**Baseline**			
Male gender	1.234	0.509–2.990	0.641
Age, years	0.999	0.971–1.028	0.948
BSA, m^2^	0.270	0.047–1.561	0.143
BMI, kg m^2^	0.912	0.821–1.013	0.085
Heart rate, min^−1^	1.015	0.996–1.034	0.133
BP systolic, mmHg	0.993	0.970–1.017	0.561
BP diastolic, mmHg	1.009	0.971–1.050	0.638
MAP, mmHg	1.000	0.966–1.035	0.996
NYHA > II	2.694	1.092–6.644	0.031
**Clinical chemistry**			
NT-proBNP, ng/L	1.162	1.072–1.260	<0.001
hs-TNT, pg/mL	1.000	0.998–1.002	0.877
**Comorbidities**			
Hypertension	1.426	0.617–3.299	0.407
Dyslipidemia	1.433	0.607–3.384	0.411
Diabetes	2.405	1.018–5.683	0.045
Renal dysfunction	0.806	0.355–1.827	0.605
**Heart catheterization**			
LVEDP, mmHg	1.066	1.028–1.107	0.001
**Echocardiography**			
IVS, mm	1.042	0.813–1.336	0.745
PW, mm	1.003	0.759–1.325	0.984
EDD, mm	1.042	0.997–1.089	0.070
ESD, mm	1.047	1.009–1.086	0.016
LV mass/BSA, g/m^2^	1.015	1.003–1.027	0.017
EDV, mL	1.005	1.001–1.009	0.008
ESV, mL	1.006	1.002–1.011	0.003
LA-Volume/BSA, mL/m^2^	1.032	1.009–1.056	0.007
E/A	1.762	1.026–3.025	0.040
E/e’	1.147	1.054–1.248	0.002
E-DT, ms	0.988	0.981–0.996	0.002
SPVF/DPVF	0.293	0.086–0.996	0.049
EF, %	0.935	0.903–0.968	<0.001
MAPSE, mm	0.813	0.721–0.917	0.001
MASV, cm/s	0.707	0.576–0.867	0.001
GLS, %	1.191	1.079–1.315	0.001
SARM, mm	0.741	0.627–0.877	<0.001
LA-VC, %	0.967	0.942–0.992	0.009

BMI, body mass index; BP, blood pressure; BSA, body surface area; DPVF, diastolic pulmonary venous flow; E/A, ratio of mitral inflow velocity (E) to atrial contraction velocity (A); E/e’, ratio of mitral inflow velocity (E) to tissue Doppler mitral annular velocity (e’); E-DT, E-wave deceleration time; EDD, end-diastolic diameter; EDV, end-diastolic volume; EF, ejection fraction; ESD, end-systolic diameter; ESV, end-systolic volume; GLS, global longitudinal strain; IVS, interventricular septum; LA-VC, left atrial volume change; LVEDP, left ventricular end-diastolic pressure; hs-TnT, high sensitive Troponin T; MAP, mean arterial pressure; MAPSE, mitral annular plane systolic excursion; MASV, mitral annular systolic velocity; NTproBNP, N-terminal pro Brain natriuretic peptide; NYHA, New York Heart Association Functional Classification; PW, posterior wall; SARM, systolic aortic root motion; SPVF, systolic pulmonary venous flow.

**Table 5:** Multivariate Cox regression analysis.

Parameter in the model	HR	CI	p value
**Model 1**			
NT-proBNP, ng/L	1.035	0.918–1.167	0.578
NYHA > II	1.601	0.559–4.587	0.381
Diabetes	1.822	0.733–4.531	0.197
SARM, mm	0.809	0.663–0.987	0.037
**Model 2**			
GLS, %	1.091	0.905–1.315	0.363
MAPSE, mm	1.069	0.832–1.374	0.603
MASV, cm/s	0.818	0.613–1.091	0.171
SARM, mm	0.831	0.628–1.100	0.196
**Model 3**			
EF, %	0.926	0.855–1.002	0.057
GLS, %	0.919	0.720–1.173	0.499
SARM, mm	0.839	0.637–1.105	0.212

GLS, global longitudinal strain; EF, ejection fraction; MAPSE, mitral annular plane systolic excursion; MASV, mitral annular systolic velocity; NTproBNP, N-terminal pro Brain natriuretic peptide; NYHA, New York Heart Association Functional Classification; SARM, systolic aortic root motion.

Reproducibility analysis revealed coefficients of variation of 5.8 for intra- and 7.6 for interobserver variability.

## Discussion

In the present study we investigated basic properties, influencing factors as well as the diagnostic and prognostic value of systolic aortic root motion (SARM) assessed by M-mode echocardiography.

### Direction of systolic aortic root motion

SARM can be described in two different ways: on the one hand in relation to the cardinal axes and planes of the body and on the other hand in relation to the main axis of the heart within the thorax.

**Figure 5:**
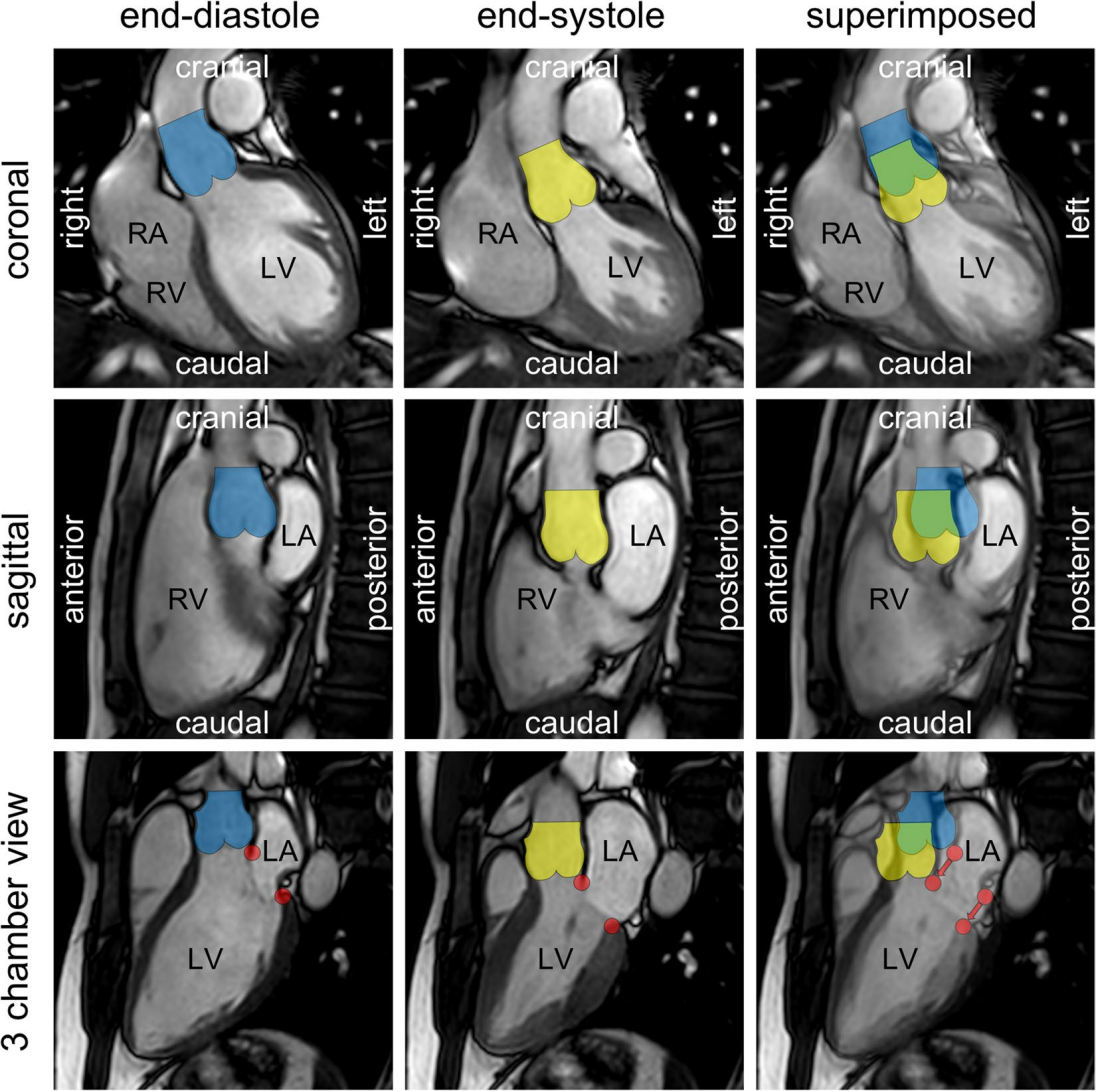
Cardiac magnetic resonance images to illustrate the position of the aortic root at different time-points during the heart cycle. Rows display the aortic root in the coronal (1^st^ row), sagittal (2^nd^ row) and an angulated plane of the left ventricular 3 chamber view (3^rd^ row). Columns represent end-diastole (1^st^ column), end-systole (2^nd^ row) and a superimposed image of the 1^st^ and 2^nd^ column. The location of the aortic root is marked blue in end-diastole and yellow in end-systole. Red dots demonstrate the displacement of the mitral anulus from end-diastole to end-systole.

With regard to the anatomical axes most former studies published on aortic root motion relied on M-mode echocardiography and described the aortic walls as pair of parallel linear signals moving anterior in systole and posterior in diastole^12–21^. This assumption is insufficient though and might be due to the fact that M-mode echocardiography is an unidimensional technique and therefore obtains signals only in one direction. However, the echo-probe is not only directed posteriorly but the imaging plane additionally has to be angulated medial and cephalic to display SARM^10^ which already indicates that a pure forward-backward motion may not completely be true. Two-dimensional B-Mode echocardiography can already display motion in 2 directions simultaneously but the restriction to specific cardiac ultrasound windows still hinders an exact alignment of SARM to the anatomic body planes. Using cardiac magnetic resonance (CMR) imaging, however, the direction of SARM was exemplary analyzed in one of the authors (MA) applying strictly orientated cine slices in the coronal and sagittal plane of the thorax. In the coronal plane the aortic root shows a downward and lateral-left displacement (Fig. 5, row 1; Supplementary Video 1), in the sagittal plane it moves downward and anterior (Fig. 5, row 2; Supplementary Video 2). Thus, the resulting vector of SARM consists of 3 components: downward, anterior and lateral which equal the motion direction of the cardiac base towards the apex during systole.

Regarding to the main axis of the heart, SARM is best visualized using cine slices of the 3 chamber view (Fig. 5, row 3; Supplementary Video 3). Here it becomes obvious, that throughout the heart cycle the aortic root parallels the motion of the mitral annulus in the longitudinal axis of the left ventricle which was already assumed by Tandon *et al*.^22^.

### Determining factors of aortic root motion

Different attempts have been made to identify potential influence factors on SARM. Pratt *et al*. correlated the amplitude of the posterior aortic wall motion with cardiac flow parameters and found the strongest relationship with LV stroke volume (r = 0.77). Thus, they concluded that SARM is a response to the action of the whole LV^12^. Keltai *et al*. and Burggraf *et al*. confirmed some of the earlier observations, even though the correlation of SARM with LV stroke volume was weaker in their studies^21,23^ (r = 0.72 and r = 0.59, respectively) as it was in ours (r = 0.57). Slightly conflicting results were reported by Rosenblatt *et al*. who found only a poor correlation^24^ (r < 0.5). While an exercise induced rise in blood pressure (BP) did not affect SARM in the study by Pratt *et al*., pharmacological lowering of BP resulted in an increase in amplitude^12^. Furthermore, the onset of SARM coincides with the rise of blood pressure and flow velocity in the ascending aorta and thus reflects the hemodynamic changes caused by LV contraction^15^. Nevertheless, no conclusive connection between mean arterial pressure and the amplitude of SARM could be established in our study (r = 0.21).

At the same time Strunk *et al*. and Biamino *et al*. offered an alternative explanation and hypothesized that posterior aortic wall motion is largely determined by left atrial (LA) volume change^13,17^. This idea has subsequently been further investigated in patients with valvular heart disease^14^. By calculating peak relative volume change between atrial diastole and systole our results reveal a moderate relationship with total excursion of the aortic root (r = 0.61). In our opinion this relationship seems logical because both, the aortic root and the LA, share a same anatomical wall. Nevertheless, this connectivity does not explain the simultaneous motion of the aorta’s root anterior wall whose motion parallels that of the posterior wall and has an even higher amplitude^12^. Except for active contraction in sinus rhythm, the atrial volume change occurs passively as a result of valvular plane displacement caused by ventricular contraction. Thus, we believe that SARM is related to but not exclusively caused by left atrial volume change and that SARM has to be attributed mainly to LV systolic function.

This could be confirmed in our study by correlating SARM to LV ejection fraction, the parameter most frequently used to grade systolic performance (r = 0.74). A comparable result was found between SARM and LV longitudinal deformation determined by global longitudinal strain (r = 0.75). This association was even higher when SARM was normalized to body surface area (r = 0.78).

### Aortic root motion in cardiac disease

SARM can be altered by different pathologic conditions. Keltai *et al*. found a reduced SARM after myocardial infarction and significant differences according to Killip classification with highest amplitude for Killip class I and lowest for Killip class IV^21^. In a study investigating various cardiac disorders Hall *et al*. described significant elevated SARM in patients with severe mitral regurgitation and atrial septal defect^16^. Similar results for mitral regurgitation were reported by Akgün *et al*.^14^ while SARM was diminished in mitral stenosis. A flattened profil of SARM was also observed by Chandraratna *et al*.^15^ in patients with hypertrophic obstructive cardiomyopathy and by Ochs *et al*.^25^ in patients with cardiac involvement of light chain Amyloidosis.

The present study investigated SARM in a prospectively recruited cohort of DCM patients and an age and gender matched control group. Like in former studies, systolic heart failure lead to a significantly reduced amplitude of total aortic root excursion compared to healthy control subjects (9 ± 3 mm vs. 12 ± 2 mm, p < 0.001). Furthermore, our results indicate that SARM is primarily driven by LV systolic function, in particular by longitudinal deformation. It is well established, that an impaired LV longitudinal function is associated with a poor prognosis in cardiac disease^3,26^ which could be confirmed by our present data. When adjusted for clinical parameters, diminished SARM is an independent predictor of cardiac death and hospitalization due to cardiac decompensation in DCM patients while a value below 7 mm was associated with worse outcome. Among multivariate models including only echocardiographic parameters, however, neither SARM nor any other measured value of longitudinal or systolic LV function could be detected as independently predictive. Although both EF and GLS are approved indicators of cardiac outcome in patients with heart failure, the contradicting information gained through the several multivariate models in our analyses might be caused by the low number of adverse events during follow-up. Furthermore, cases in which it is impossible to calculate EF and/or GLS are rare, especially in times of echocardiography contrast agents. This might limit the use of SARM as a general index of systolic LV function.

## Limitations

First, this was a monocentric study conducted with a specific echocardiographic machine for image acquisition and its proprietary software package was used for analysis. Second, due to the divergent acoustic window, the displacement axis of SARM may not be completely concordant with the global longitudinal motion of the LV. Thus, compared to speckle tracking echocardiography SARM serves only as a surrogate parameter of global longitudinal LV deformation and is not suitable to determine regional abnormalities. Third, discrepancy in information obtained by multivariate models in our analyses might be caused by the low number of adverse events during follow-up.

## Conclusion

Due to its high echogenicy, SARM can easily be visualized by M-mode echocardiography. Contrary to many previous reports we demonstrated that SARM is not exclusively directed anterior but shows an additional downward and lateral-left displacement similar to the systolic movement of the cardiac base towards the apex.

Abnormal SARM is a frequent finding in cardiac disease. Alterations are, however, not specific to a particular pathology but can generally be regarded as a prognostically unfavorable sign in patients with systolic heart failure.

Our data suggest that SARM is closely related to global longitudinal strain and thus might represent an alternative measure of LV longitudinal function especially when strain assessment is not feasible or available.

## Methods

### Study population

The present study is part of the project “New echocardiographic parameters for assessment of longitudinal left ventricular function” (ClinicalTrials.gov Identifier: NCT01275963). A corresponding recruitment strategy, inclusion and exclusion criteria as well as the follow-up process have therefore already been described in detail previously^27^.

Initial analysis for the current study is based on data derived from a recent trial and included the complete cohort of 202 patients with dilated cardiomyopathy who were recruited between January 2009 and December 2015 in our cardiology department and the same number of age and gender matched control subjects. Digitally stored echocardiographic examinations were screened for availability of SARM measurements and all suitable datasets have subsequently been re-matched for age and gender. The final study population consisted of 180 patients and 180 healthy controls.

This study was carried out after approval by the ethics committee of the University of Heidelberg in concordance with the Declaration of Helsinki. Written informed consent was provided by all participants of this study.

### Echocardiography

Echocardiography was conducted with a commercially available ultrasound machine (Vivid E9 BT 11; GE Vingmed Ultrasound, Horten, Norway) using a 1.5- to 4.6-MHz phased-array probe (M5S-D). The sampling rate was adjusted to 55 to 60 frames/sec for B-mode, M-mode was recorded at 1.000 to 2.000 frames/sec. Images from 3 consecutive heart cycles were acquired and stored digitally in RAW-DICOM format for later offline analysis. Measurements were performed according to the recommendation of the American Society of Echocardiography and the European Association of Cardiovascular Imaging^28^ using commercially available software (EchoPAC version 110.1.1 BT 11; GE Vingmed Ultrasound). Global longitudinal strain (GLS) was determined using the embedded Automated Function Imaging tool.

SARM was displayed starting from a left parasternal B-mode short axis view of the left ventricle with the echo probe directing posteriorly. The imaging plane was then tilted medial and angulated cephalic until the opening and closing of the aortic valve was visible. Finally, the M-mode beam was placed through the center of the aortic roots cross section (Figs 1 and 2).

### Statistical analysis

Statistical data analysis was performed using SPSS version 24 (IBM Corporation, Armonk, New York). Continuous variables are presented as mean ± SD or as median with interquartile range as appropriate. Values were compared with Student’s t test for normally distributed data, otherwise the Wilcoxon signed rank test for paired observations (patients vs control subjects) or the Mann-Whitney-Wilcoxon test for unpaired observations (patients with or without events) was used. Categorical values are expressed as number (percentage) and were compared using χ2 or McNemar depending on data distribution. All analyses were of explorative nature. A p-value of < 0.05 was denoted statistically significant.

Association between SARM and potential influence factors was explored by Pearson’s correlation and linear regression analysis. The ability of SARM to discriminate patients with DCM from healthy individuals as well as to discriminate patients with from those without a cardiac event was tested using receiver operating characteristic analyses. Cutoff values were calculated using Youden’s index.

To evaluate the prognostic value of SARM a combined end-point consisting of cardiovascular death^29^ and hospitalization for heart failure was defined. The occurrence of events over time is displayed by Kaplan-Meier curves. Groups were compared using the Log-rank test. Univariate Cox regression was used to calculate hazard ratios of clinical and echocardiographic variables. Parameters with significant impact on patient outcome were put into different multivariate models to identify independently prognostic factors. Hazard ratios with 95% confidence intervals and p-values are provided. According to the number of events in this study and with the intention not to overfit the analysis, multivariate models were restricted to a maximum of 4 variables.

Reproducibility of SARM measurements was tested by reanalyzing 20 randomly chosen patients and control subjects in a blinded fashion by the same and by a second experienced investigator. Intra- and interobserver variability results are expressed as coefficients of variation.

The datasets generated and analyzed during the current study are available from the corresponding author on reasonable request.

## Supplementary information

Supplementary figure S1

Supplementary video 1

Supplementary video 2

Supplementary video 3
