# Supplementary material for: Pathophysiological background and prognostic implication of systolic aortic root motion in non-ischemic dilated cardiomyopathy
Source: Sci Rep. 2019 Mar 7;9:3866. doi: 10.1038/s41598-019-40386-z (PMC6405754; doi:10.1038/s41598-019-40386-z)
Supplement: Supplementary file 1 — Supplementary figure S1 [file 41598_2019_40386_MOESM1_ESM.pdf]

Pathophysiological background and prognostic implication of systolic aortic root motion in non-ischemic dilated cardiomyopathy

Matthias Aurich, Matthias Niemers, Patrick Fuchs, Sebastian Greiner, Matthias Müller-Hennessen, Lorenz Uhlmann, Evangelos Giannitsis, Philipp Ehlermann, Benjamin Meder, Hugo A. Katus, Derliz Mereles

Supplementary figure S1:

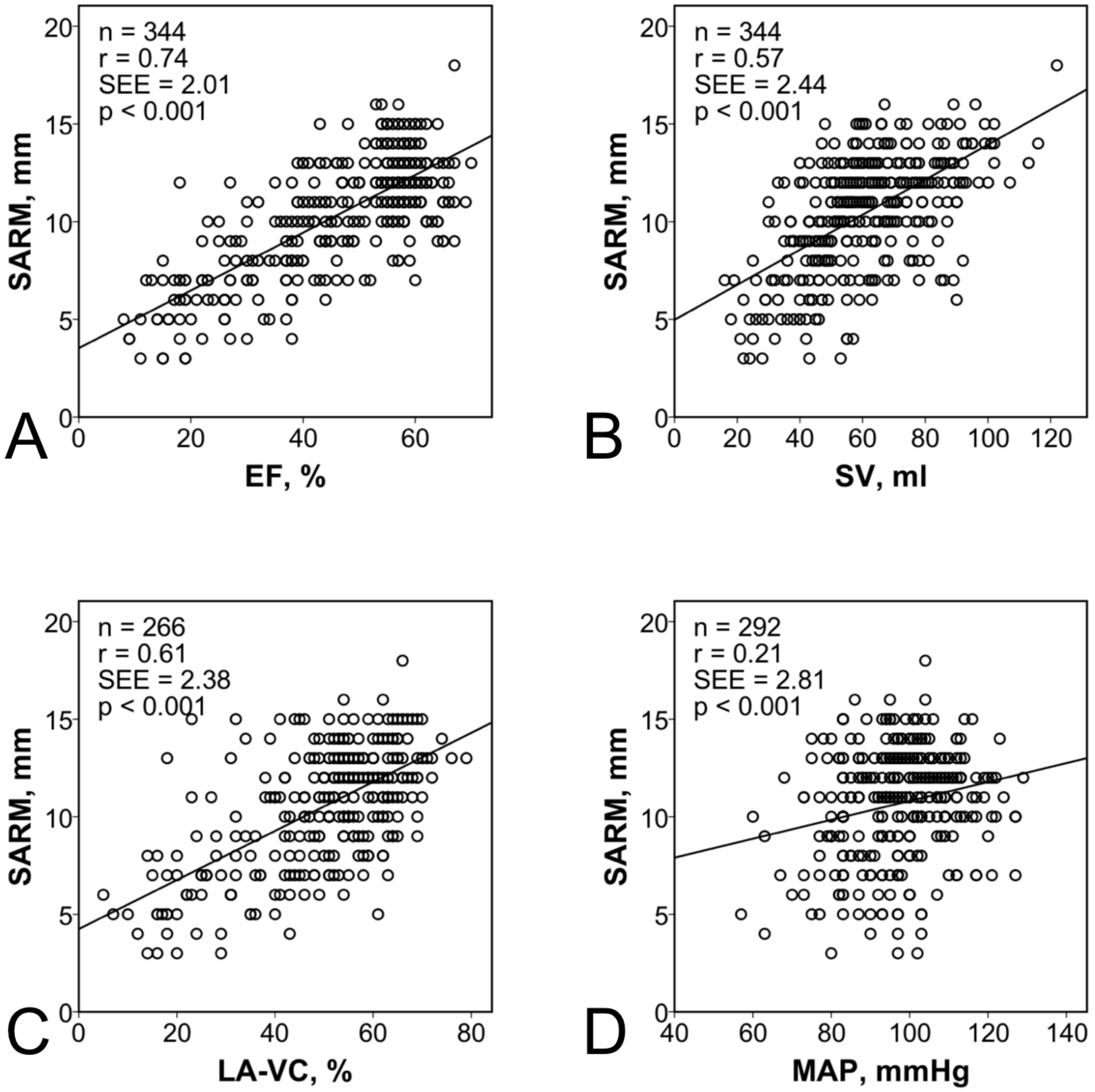

Correlations between systolic aortic root motion (SARM) and ejection fraction (EF), stroke volume (SV), left atrial volume change (LA-VC) and mean arterial pressure (MAP). SEE, standard error of estimate.
